# Acute ischemic stroke patients with diffusion-weighted imaging-Alberta Stroke Program Early Computed Tomography Score ≤ 5 can benefit from endovascular treatment: a single-center experience and literature review

**DOI:** 10.1007/s00234-019-02177-1

**Published:** 2019-02-06

**Authors:** Kangping Song, Min Guan, Wenxian Li, Zhen Jing, Xiaomei Xie, Changzheng Shi, Jianye Liang, Hongyu Qiao, Li’an Huang

**Affiliations:** 1 Department of Neurology, Clinical Neuroscience Institute, The First Affiliated Hospital, Jinan University, Guangzhou, Guangdong China; 2 Medical Imaging Center, The First Affiliated Hospital, Jinan University, Guangzhou, Guangdong China

**Keywords:** DWI-ASPECTS, Endovascular treatment, Ischemic stroke, Prognosis

## Abstract

**Purpose:**

The recommendation strength of the guidelines for mechanical thrombectomy among patients with large pre-treatment core infarct is weak. We evaluated the safety and outcome of endovascular treatment for acute ischemic stroke with diffusion-weighted imaging-Alberta Stroke Program Early Computed Tomography Score (DWI-ASPECTS) ≤ 5.

**Methods:**

Data on acute ischemic stroke patients with DWI-ASPECTS ≤ 5 who underwent endovascular treatment within 6 h, or presented an arterial spin labeling-DWI (ASL-DWI) mismatch within 12 h, at our center were retrospectively collected. We report the clinical characteristics and outcome of every patient, and review the relevant literature.

**Results:**

Among the 19 patients who were enrolled, all experienced successful reperfusion, and 10 achieved a favorable outcome (modified Rankin scale (mRS) ≤ 2). Two patients presented with symptomatic intracranial hemorrhage (sICH); both of them had a poor outcome (mRS > 2).

**Conclusion:**

Acute ischemic stroke patients with large DWI lesions caused by large vessel occlusion can achieve a favorable clinical outcome with endovascular treatment if recanalization is performed within 6 h, or after 6 h in case of an ASL-DWI mismatch.

**Electronic supplementary material:**

The online version of this article (10.1007/s00234-019-02177-1) contains supplementary material, which is available to authorized users.

## Introduction

With 5.9 million deaths and 102 million disability-adjusted life years (DALYs) lost, stroke is the second leading cause of death and the third leading cause of DALYs lost worldwide [1]. Large infarctions account for 7.6% of all ischemic strokes [2] and correlated with a high rate of mortality [3]. Intravenous thrombolysis (IVT) is a generally accepted effective therapy for acute ischemic stroke (AIS), but it has a low rate of recanalization for large vessel occlusion (LVO). In the EXTEND-IA TNK trial, only 10% of patients with LVO reached successful reperfusion after receiving rt-PA [4]. In recent years, a series of multi-center randomized clinical trials (MR CLEAN, EXTEND-IA, REVASCAT, SWIFT PRIME, and ESCAPE) [5–9] showed that mechanical thrombectomy with a stent retriever improves the functional outcome of patients with ischemic stroke caused by proximal vessel occlusion, compared with IVT alone, leading to neurointervention in combination with IVT as a first-line therapy for acute LVO in the anterior circulation. The representative, multi-center clinical trials [7–9] used massive cerebral infarction as an exclusion criterion, with the result of endovascular treatment (EVT) being deemed unsuitable for treatment, or having “uncertain benefits,” in these cases, according to an update in the endovascular treatment guidelines in Europe and the USA [10, 11]. The latest 2018 AHA/ASA guidelines for AIS recommend mechanical thrombectomy for ASPECTS ≥ 6 cases while its benefits for cases with ASPECTS < 6 were considered uncertain [12]. Many studies have confirmed that despite early successful recanalization, patients with large infarction barely benefited from EVT [13–16]. In contrast to previous studies, a recent small-sample study showed that young patients with large infarct lesions could benefit from EVT [17]. Similarly, Inoue et al. [18] analyzed patients of all ages and found that selected patients with AIS with massive DWI lesions (DWI-ASPECTS < 5) might still benefit from EVT if a successful recanalization is achieved. Most recently, Gautheron et al. [19] reported that stroke patients with large DWI lesions of > 70 mL may benefit from reperfusion therapy. Due to the disagreements in the results of previous research, there is no consistent conclusion regarding whether EVT should be performed or withheld for patients with large infarction lesions.

The ASPECTS is primarily used in cranial computed tomography (CT) to assess the extent of acute cerebral infarction, with a value range from 0 to 10, with lower scores indicating a larger infarct size [20]. Nezu et al. [21] reported that ASPECTS based on DWI were about 1 point lower than those based on CT, and that both DWI-ASPECTS and CT-ASPECTS were predictors of clinical outcomes, while another study considered the DWI-ASPECTS of better sensitivity and predictive ability than the CT-ASPECTS [22]. Inoue et al. [18] suggested DWI-ASPECTS < 5 as the threshold of unfavorable prognosis for patients receiving EVT, while Han et al. [14] suggested DWI-ASPECTS ≤ 3 as the threshold. Meanwhile, another study [23] revealed that there was no difference in the prognosis between patients with DWI-ASPECTS of 4–6 and 7–10. However, recently, Manceau et al. [24] examined outcomes of ischemic stroke patients with DWI-ASPECTS ≤ 5 and found that a DWI-ASPECTS > 2 was a pretreatment marker of favorable neurological outcomes.

Due to the continued uncertainty of the efficacy of EVT in patients with low DWI-ASPECTS, we conducted this retrospective single-center study to examine the outcome of EVT in patients with pretreatment DWI-ASPECTS ≤ 5 and reviewed published studies in patients with large infarct volumes to determine if large infarctions should be a contraindication for EVT.

## Materials and methods

### Single-center series

#### Study population

**Fig. 1:**
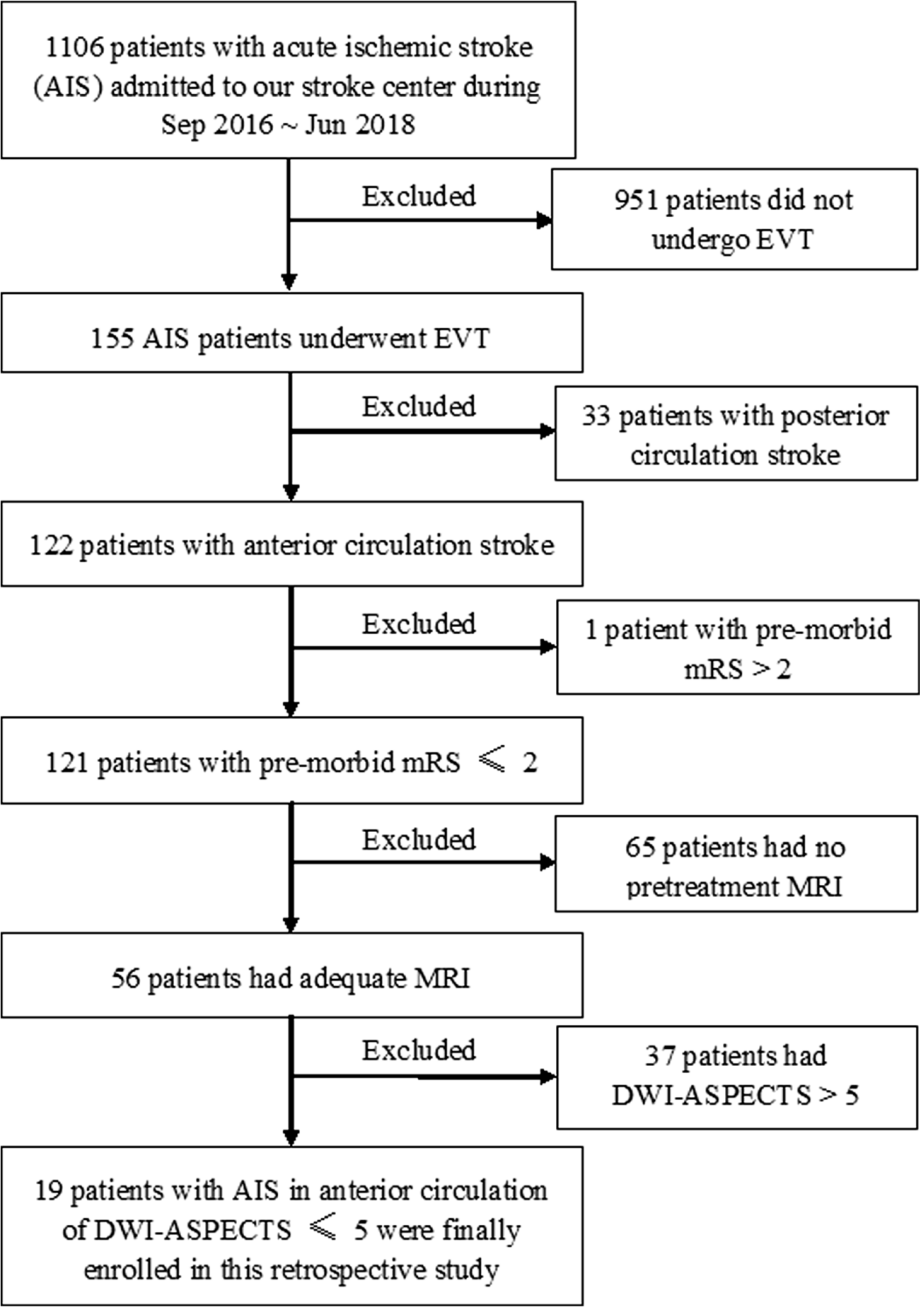
Flow chart of patient screening

We retrospectively reviewed all patients with acute anterior circulation infarcts, as identified by MRI and digital subtraction angiography (DSA), who received EVT at the First Affiliated Hospital of Jinan University from September 2016 to June 2018. The inclusion criteria for participants were the following: acute occlusion of the internal carotid artery (ICA) or the middle cerebral artery (MCA); DWI-ASPECTS ≤ 5; and underwent IVT followed by EVT or direct EVT. Individuals who rejected endovascular intervention, had a DWI-ASPECT > 5, had a posterior circulation infarct, or had a pre-morbid modified Rankin scale (mRS) > 2 were excluded from this study. A flow chart of the patient screening procedure is shown in Fig. 1.

#### Imaging protocols

MR examinations were conducted using a Discovery 750 3.0-Tesla scanner with an 8-channel head coil (GE Healthcare, Milwaukee, WI, USA). All patients underwent MRI scanning prior to EVT. The standard imaging protocol included DWI, 3D time-of-flight magnetic resonance angiography (3D TOF MRA), and 3D arterial spin labeling (ASL). Follow-up imaging was performed using CT or MRI at 24 h after intervention. The imaging parameters for DWI were as follows: TR 5200 ms, TE 87.4 ms, *b* values 0 s/mm^2^ and 1000 s/mm^2^, matrix 160 × 160, FOV 24 × 24 cm, and slice thickness 5 mm with a 1-mm gap. The 3D TOF MRA acquisition parameters were TR 23 ms, TE 2.8 ms, matrix 256 × 192, FOV 22 × 16.5 cm, and slice thickness 1.4 mm with no gap. The 3D ASL acquisition parameters were TR 4564 ms, TE 11 ms, post-labeling delay 1525 ms, matrix 128 × 128, FOV 24 × 24 cm, excitation 3, and slice thickness 4 mm with no gap.

#### Acute stroke treatment

For acute ischemic stroke treatment, EVT (mechanical thrombectomy or extracranial ICA stent implantation) alone, or in combination with IVT, was used to obtain fast recanalization and reperfusion. Patients who met the criteria for IV rt-PA within 4.5 h of symptom onset were treated with 0.9 mg/kg IV rt-PA, unless the family rejected treatment. If a LVO was confirmed by MRA, EVT was performed in cases where the time of symptom onset was within 6 h or when an obvious mismatch of ASL-DWI was detected within 12 h. The definition of an ASL-DWI mismatch was based on a previous study, specifically, on a ratio of hypoperfusion tissue and ischemic core ≥ 1.8 [25]. For mechanical thrombectomy, the Solitaire stent system (Solitaire, Micro Therapeutics Inc. DBA ev3 Neurovascular, CA, USA) was used to retrieve the embolus, and we used the Wallstent™ (Boston Scientific Corporation, Galway, Ireland) for treating extracranial ICA stenosis.

#### Imaging analysis

Pretreatment ASPECTS were evaluated by two independent neuroradiologists, blinded from the study protocol and clinical information, on the basis of baseline DWI. The infarction volume was semi-automatically measured by manual outlining of hyper-intense lesions on DWIs by two readers using the Image Processing Analysis in Java software (ImageJ, version 1.46r). Because our DWI sequence was acquired with a gap of 1 mm, the spacer infarct area was defined as the mean area between two adjacent layers. The total infarction volume was calculated as the total hyper-intense area in every slice multiplied by the thickness of the scanned layer and gap. Hypoperfusion lesions were segmented manually, slice by slice, and calculated automatically by multiplying the total slice thickness by the lesion area on cerebral blood flow maps, which were generated by commercially available scanner software (Functool 3D ASL, Software version 4.5, GE Medical Systems, Milwaukee, WI, USA). Good reperfusion was defined as a thrombolysis in cerebral infarction (TICI) grade of 2b or 3 [26]. When there was disagreement in assessment, a consensus conference was held to draw a conclusion.

#### Clinical assessment

The National Institutes of Health Stroke Scale (NIHSS) was used to assess the neurologic deficits at baseline and 24 h after EVT. Early neurological improvement was defined as a decrease of 8 or more points on the NIHSS or a score of 0 to 2 at 24 h after intervention [7]. Early neurological deterioration was defined as an increase of 4 or more point in the NIHSS score within 24 h after intervention [27, 28]. A 3-month post-stroke mRS was used to evaluate the clinical outcome. A favorable outcome was defined as mRS ≤ 2, and a poor outcome was defined as mRS > 2. Symptomatic intracranial hemorrhage (sICH) after treatment was defined as any apparent sign of hemorrhage detected by CT or MRI at 24 h after treatment, with an increase in the NIHSS score of 4 or more attributable to ICH [29].

#### Data analyses

To determine the difference between patients who experienced either a favorable or unfavorable outcome, normally distributed continuous variables, represented as mean ± standard deviation (SD), were analyzed using unpaired *t* tests; not normally distributed continuous variables were represented as medians (interquartile range [IQR]) and analyzed using the Mann-Whitney *U* test. Ordinal categorical variables were represented as medians (IQR) and analyzed using the Mann-Whitney *U* test. Unordered categorical variables were represented as numbers (percentages) and chi-square tests or Fisher’s exact test was used for analysis. We also assessed the outcome and safety of bridging therapy (IVT followed by EVT) and EVT alone, using Fisher’s exact test. All statistical analyses were performed using IBM SPSS 19.0 software. Statistical significance was defined as *P* < 0.05.

### Literature review

We searched PubMed, Embase, and Web of Science databases using the terms “stroke,” “cerebral infarct,” “infarction,” “endovascular treatment,” “embolectomy,” “thrombectomy,” “neuroendovascular interventions,” “neurointervention,” “volume,” and “ASPECT,” with no restrictions regarding the publication date. Two authors were responsible for identifying relevant studies by scanning through titles and abstracts. Studies that performed EVT and involved patients with AIS and DWI-ASPECTS ≤ 5 were selected for a full-text review.

## Results

### Single-center series

**Fig. 2:**
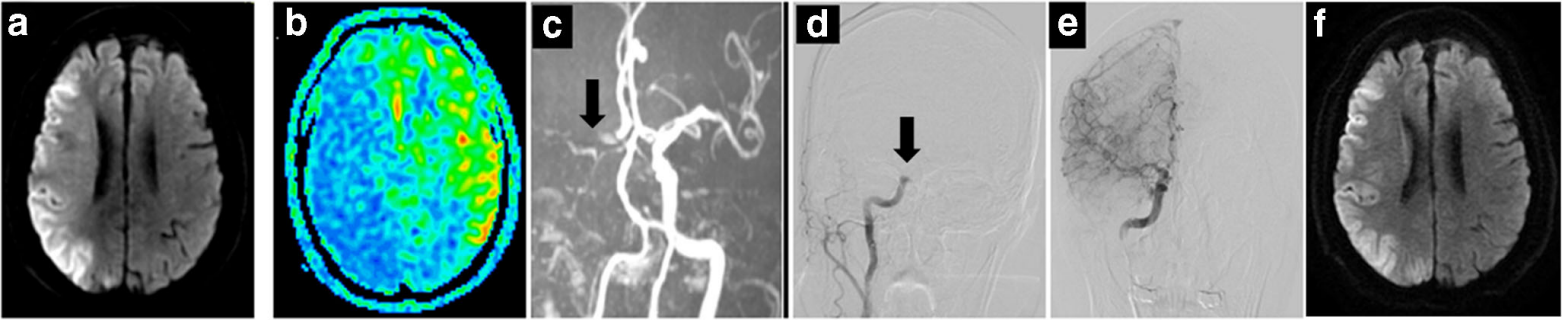
A 85-year-old man (NO. 3 in Supplementary Tables 1 and 2) with a DWI-ASPECTS of 2 and an ASL-DWI mismatch ratio of 1.95, received bridging therapy and obtained successful reperfusion at 155 min from onset. This patient achieved a favorable outcome as 3 months (mRS = 2). **a** Infarction lesion presented on DWI (shown as high signal). **b** Hypoperfusion area shown on ASL-CBF map (blue). **c** Right ICA occlusion shown on MRA (black arrow). **d** DSA showing occlusion site of right ICA (black arrow) before EVT. **e** DSA showing reperfusion of occlusion vessel after EVT. **f** DWI image at 24 h after EVT

**Fig. 3:**
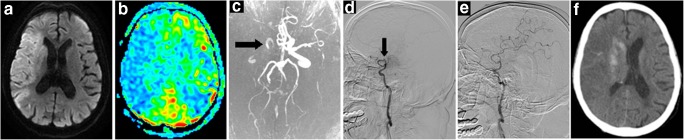
A 82-year-old woman (NO. 17 in Supplementary Tables 1 and 2) with a DWI-ASPECTS of 5 and an ASL-DWI mismatch ratio of 1.38 was treated by EVT alone and obtained successful reperfusion at 325 min from onset. This patient died of cerebral edema and herniation 10 days after intervention. **a** Infarction lesion presented on DWI (shown as high signal). **b** Hypoperfusion area shown on ASL-CBF map (blue). **c** Right ICA-MCA occlusion shown on MRA (black arrow). **d** DSA showing occlusion site of right ICA-MCA (black arrow) before EVT. **e** DSA showing reperfusion of occlusion vessel after EVT. **f** CT image at 24 h after EVT

Nineteen eligible patients were recruited for this study, 14 of which were male. The mean age was 64.9 ± 14.1 years (range, 39–85). All patients had a baseline DWI-ASPECTS ≤ 5, and the mean infarction volume was 145 ± 95.0 mL. Twelve patients were treated with EVT within 6 h, but in three of them, information regarding an ASL-DWI mismatch could not be obtained. In two cases, no CBF maps were obtained due to head motion, and in one case, ASL was not performed. For one of seven patients who received treatment within 12 h, we could not obtain a mismatch ratio due to head motion and poor ASL imaging quality. We included this patient despite the missing mismatch ratio information, because he presented a large lesion infarction and accepted EVT within 12 h, and thus met the inclusion criteria. The median admission NIHSS score was 16 (15–18). Seven of 19 patients had an ICA occlusion, 10 had an MCA occlusion, and two patients had a coexistent lesion of the ICA and the MCA. Bridging therapy was performed in eight patients, while the other 11 patients underwent EVT only. All patients achieved successful reperfusion. Five of the included patients experienced significant neurological improvements at 24 h after intervention, and all of them presented a favorable outcome of mRS ≤ 2 at 3 months; all three patients who showed early neurological deterioration presented a poor outcome ultimately. Overall, 10 patients (52.6%) showed good neurological outcomes at 3 months. sICH was observed in two patients, and one of them subsequently passed away during the hospital stay, due to ICH and brain swelling complications. The mortality rate in this study was 15.8% (3/19). Other two patients died during the course of this study, one due to a severe infection during hospitalization, and the other due to cerebral edema and herniation. Baseline epidemiological risk factors for stroke and demographic data are depicted in Supplementary Table 1. Detailed information regarding pretreatment, follow-up of neurological functions, imaging, and serious complications are shown in Supplementary Table 2. Representative images of two patients, including DWI, ASL, MRA, and DSA, both before and after EVT, and MRI or CT images 24 h post treatment are shown in Figs. 2 and 3, respectively.

We analyzed all potential risk factors of poor clinical outcome, and no differences were found between patients with favorable and poor outcomes, except for age and NIHSS after 24 h of EVT (Table 1). The safety and outcome of bridging therapy and EVT alone did not significantly differ (*P* = 0.170; *P* = 1.00, respectively).

**Table 1:** Comparison among patients who had favorable outcomes (mRS ≤ 2) and poor outcomes (mRS > 2) at 3 months

Characteristic	Favorable outcome (*n* = 10)	Poor outcome (*n* = 9)	*P* value
Age (years)	58.3 ± 14.2	72.2 ± 10.2	0.027*
Male	7 (70.0%)	7 (77.8%)	1.00
Hypertension	5 (50.0%)	7 (77.8%)	0.35
DM	1 (10.0%)	3 (33.3%)	0.303
AF	4 (40.0%)	3 (33.3%)	1.00
CHD	2 (20.0%)	2 (22.2%)	1.00
Smoking	1 (10.0%)	3 (33.3%)	0.303
IS	1 (10.0%)	2 (22.2%)	.582
Systolic pressure	129 ± 24.1	139 ± 24.8	0.426
Diastolic pressure	75.6 ± 15.9	75.7 ± 16.6	0.995
Glucose	6.54 ± 1.09	8.52 ± 3.13	0.103
Right side lesion	6 (60.0%)	6 (66.7%)	1.00
Intravenous rt-PA	6 (60.0%)	2 (22.2%)	0.170
Time from onset to intravenous rt-PA (min)	102 ± 22.4	90.0 ± 28.3	0.565
Time from onset to groin puncture (min)	325 ± 195	314 ± 156	0.897
Time from onset to reperfusion (min)	400 ± 207	388 ± 168	0.889
DWI-ASPECTS	4.5 (2–5)	4 (4–5)	0.571
Infarct volume	141 ± 97.9	149 ± 97.4	0.863
Baseline NIHSS	15.5 (14.25–18.25)	16 (15–18)	0.649
24-h NIHSS	9.5 (5–14)	17 (12.5–30)	0.005**
TICI			1.00
2b	3 (30.0%)	2 (22.2%)	
3	7 (70.0%)	7 (77.8%)	
sICH	0 (0%)	2 (22.2%)	0.211

*DM*, diabetes mellitus; *AF*, atrial fibrillation; *CHD*, coronary heart disease; *IS*, history of ischemic stroke

### Literature review

Information from six qualified, published studies, including a total of 369 eligible patients with DWI-ASPECTS ≤ 5 who received EVT, were collected; the details of this selection are presented in Table 2.

**Table 2:** Summary of the published literature on the use of EVT in anterior circulation stroke with DWI-ASPECTS ≤ 5

Study	DWI-ASPECTS range		Sample size	Mean age (years)	Baseline NIHSS	Combined with IVT	Device	Reperfusion rate	Favorable outcome rate	Hemorrhagic transformation^‡^	Death rate
Manceau et al. 2018 [24]	0–5		82	64.6 ± 14.4	18.4 ± 5.4	54 (66%)	Stent retrievers or aspiration methods	41 (50%)	28 (34%)	19 (23%)	30 (37%)
Inoue et al. 2014 [18]	0–5		75	67.7 ± 17.7	18 (16–22)	48 (64%)	Stent retrievers	NK	20 (26.7%)	12 (16%)	36 (48%)
Deguchi et al. 2014 [30]	0–5	< 3 h	25	68 ± 11	19 (9–38)	12 (48%)	NK	25 (100%)	7 (28%)	5 (20%)	7 (28%)
		3–8 h	7	73 ± 12	21 (14–28)	0	NK	7 (100%)	2 (29%)	0 (0%)	1 (14%)
Kim, et al. 2016 [23]	4–5		22	NK	NK	NK	Stent retrievers or aspiration methods	NK	10 (45.5%)	NK	NK
Desilles, et al. 2017 [16]	0–5		132	NK	NK	NK	Stent retrievers or aspiration methods	87 (65.9%)	31/123 (25.2%)^†^	75/125 (60%)^†^	46 (37.4%)^†^
Daniere, et al. 2015 [31]	0–4		26	NK	17.24	11 (42%)	Stent retrievers	21 (80%)	9 (34.6%)	NK	NK

*NK*, not known

^†^The result of rate was not consistent with the sample size due to missing data presented in this trial

^‡^Some studies refer to sICH; some refer to PH2 or any hemorrhage

## Discussion

Our results revealed that some patients with large cerebral infarcts could achieve favorable outcomes with EVT if they received treatment within 6 h of symptom onset or presented with an obvious mismatch of ASL-DWI within 12 h. In the current study, all patients achieved successful reperfusion, and 10 patients (52.6%) presented good neurological outcomes at 3 months. By reviewing published studies [16, 18, 23, 24, 30, 31], we found that about 66.5% of patients with low DWI-ASPECTS achieve successful recanalization and approximately 29.7% can be functionally independent, which is superior to the outcome of traditional medical treatment (i.e., less than 20%) [3, 32]. Salvageable penumbra tissue is a key factor for favorable prognosis; this has been confirmed by a large number of studies [25, 33–35]. A recent study [36] suggested that the response to EVT in patients with AIS depends on salvageable tissue but not time from stroke onset. By restricting our patient selection to a 6-h time window since stroke onset, or to an apparent ASL-DWI mismatch in situations where the time window was exceeded, our center obtained encouraging results from EVT for large cerebral infarctions. These findings may be helpful for the selection of appropriate patients for EVT in the future; due to the limited sample size, however, it is too early to generalize our results.

Among the 10 patients with good functional outcomes, five showed significant early neurological improvements on the NIHSS at 24 h. Unfortunately, all three patients who experienced neurologic deterioration at 24 h after intervention had a worse final neurological outcome. This may indicate that neurological improvement or deterioration during the first 24 h is the most important factor for the prediction of clinical outcome, which is consistent with the previous published study [37]. When we tested potential risk factors for poor outcomes, we found no statistically significant differences between patients with good or poor outcome for baseline NIHSS, DWI-ASPECTS, and time from symptom onset to reperfusion; only for age and NIHSS 24 h after intervention, such differences were observed, which emphasizes the importance of early neurological changes. Pu et al. [38] came to the same conclusion, namely that 24-h neurological improvement is an independent predictor for outcome. In a study carried out by Daniere et al. [31], 60% of patients with large infarctions who were younger than 70 years could obtain a favorable outcome, while only 10% of elderly patients achieved good outcomes. Therefore, we suggest that age should be considered when selecting patients with large infarction lesions for EVT.

Studies in recent years [39, 40] have added support to the principle that a longer time prior to reperfusion might relate to poor clinical outcome. On the other hand, Lansberg et al. [36] concluded that if salvageable tissue existed, patients’ functional outcomes were not time-dependent after endovascular reperfusion. Most recently, in the DAWN clinical trials, thrombectomy was performed in patients with clinical deficits and infarct volume mismatches 6–24 h after stroke, which resulted in a significantly superior outcome compared with standard care [41], and these findings were supported by the multi-center trial DEFUSE3, which demonstrated that EVT combined with standard medical therapy was better than standard medical therapy alone if patients were treated within 6 to 16 h from onset [42]. While the results of our small-sample study are consistent with those of previous studies, showing that reperfusion time does not influence clinical outcome, our data does not allow to draw a clear conclusion due to the limited number of subjects. The finding that baseline NIHSS also had no effect on clinical outcome may be explained by two reasons: the inclusion criterion of large infarction lesions and the small sample size of this study.

Despite a large number of studies utilizing the ASPECTS system and the various thresholds set for predicting clinical outcomes, we found no difference in DWI-ASPECTS between patients with good and those with poor prognosis in our study. A potential reason for this finding may be that the inclusion criteria of only DWI-ASPECTS ≤ 5 led to relatively high homogeneity of the included patient group, and the small sample size may have further contributed to the lack of difference. It is worth noting that the patient in our center with the lowest DWI-ASPECTS value who reached a favorable outcome had a score of 1; this suggests that DWI-ASPECTS may not be a predictive factor for prognosis, which differs markedly from previous research.

sICH occurred in two patients at our center, and both of them showed significantly worse neurological outcomes. Previous published study demonstrated that intracerebral hemorrhage is a serious complication in the application of recanalization treatment and is closely associated with the prognosis of patients with AIS [43]. How to avoid hemorrhage after recanalization is a difficult problem to be solved.

Three of the six reviewed studies reported that successful reperfusion is associated with good prognosis [16, 18, 24]. These studies suggest that low ASPECTS should not be a contraindication for EVT treatment. However, there were some shortcomings in these cited studies. Most importantly, not all studies enrolled patients with DWI-ASPECTS of 0~5, as one study [23] excluded patients with DWI-ASPECTS ≤ 3 and another study [31] provided information for a subgroup with DWI-ASPECTS < 5 only. Besides, as some of the data were extracted from a subgroup, not a separate group in the original analysis, information regarding some of the risk factors (e.g., age, pretreatment NIHSS, reperfusion rate) that may affect prognosis is incomplete. In addition, we did not analyze hemorrhage rates, due to different study standards, as some included all hemorrhagic events [16] while others included parenchymal hematoma type 2 [24] or sICH only [18, 23, 30].

Overall, in view of previously published and current data regarding EVT for patients who have suffered large cerebral infarctions, we concluded that cases with DWI-ASPECTS ≤ 5 should not be withheld from EVT, considering that about 30% of patients with AIS can benefit from EVT; this proportion might be higher if a high level of successful reperfusions can be achieved. Salvageable penumbra tissue may facilitate favorable prognosis.

This study has several limitations. First, the sample size is very small, which makes selection biases very likely. Second, the retrospective nature of our study and the lack of a high-quality, randomized control group, restrict extrapolation of our results. Third, the heterogeneity of the patient group due to the inclusion criteria and missing information in the literature review prevented us from conducting a statistical analysis on probable influencing factors of EVT for patients with large infarction lesions.

## Conclusion

Patients with large-volume infarctions caused by large vessel occlusion could obtain favorable clinical outcomes with EVT within 6 h of onset or after 6 h if presenting with an ASL-DWI mismatch. Successful reperfusion may yield better neurological outcomes in patients with large infarction lesions who had large vessel occlusion. For further evaluation, randomized controlled studies with larger sample sizes are required.

## Electronic supplementary material

ESM 1 (PDF 171 kb)

## References

[1] Hankey GJ (2017). Stroke. Lancet.

[2] Heinsius T, Bogousslavsky J, Van Melle G (1998). Large infarcts in the middle cerebral artery territory. Etiology and outcome patterns. Neurology.

[3] Rieke K, Schwab S, Krieger D, von Kummer R, Aschoff A, Schuchardt V, Hacke W (1995). Decompressive surgery in space-occupying hemispheric infarction: results of an open, prospective trial. Crit Care Med.

[4] Campbell BCV, Mitchell PJ, Churilov L, Yassi N, Kleinig TJ, Dowling RJ, Yan B, Bush SJ, Dewey HM, Thijs V, Scroop R, Simpson M, Brooks M, Asadi H, Wu TY, Shah DG, Wijeratne T, Ang T, Miteff F, Levi CR, Rodrigues E, Zhao H, Salvaris P, Garcia-Esperon C, Bailey P, Rice H, de Villiers L, Brown H, Redmond K, Leggett D, Fink JN, Collecutt W, Wong AA, Muller C, Coulthard A, Mitchell K, Clouston J, Mahady K, Field D, Ma H, Phan TG, Chong W, Chandra RV, Slater LA, Krause M, Harrington TJ, Faulder KC, Steinfort BS, Bladin CF, Sharma G, Desmond PM, Parsons MW, Donnan GA, Davis SM, Investigators E-IT (2018). Tenecteplase versus alteplase before thrombectomy for ischemic stroke. N Engl J Med.

[5] Berkhemer OA, Fransen PS, Beumer D, van den Berg LA, Lingsma HF, Yoo AJ, Schonewille WJ, Vos JA, Nederkoorn PJ, Wermer MJ, van Walderveen MA, Staals J, Hofmeijer J, van Oostayen JA, Lycklama A, Nijeholt GJ, Boiten J, Brouwer PA, Emmer BJ, de Bruijn SF, van Dijk LC, Kappelle LJ, Lo RH, van Dijk EJ, de Vries J, de Kort PL, van Rooij WJ, van den Berg JS, van Hasselt BA, Aerden LA, Dallinga RJ, Visser MC, Bot JC, Vroomen PC, Eshghi O, Schreuder TH, Heijboer RJ, Keizer K, Tielbeek AV, den Hertog HM, Gerrits DG, van den Berg-Vos RM, Karas GB, Steyerberg EW, Flach HZ, Marquering HA, Sprengers ME, Jenniskens SF, Beenen LF, van den Berg R, Koudstaal PJ, van Zwam WH, Roos YB, van der Lugt A, van Oostenbrugge RJ, Majoie CB, Dippel DW, Investigators MC (2015). A randomized trial of intraarterial treatment for acute ischemic stroke. N Engl J Med.

[6] Campbell BC, Mitchell PJ, Kleinig TJ, Dewey HM, Churilov L, Yassi N, Yan B, Dowling RJ, Parsons MW, Oxley TJ, Wu TY, Brooks M, Simpson MA, Miteff F, Levi CR, Krause M, Harrington TJ, Faulder KC, Steinfort BS, Priglinger M, Ang T, Scroop R, Barber PA, McGuinness B, Wijeratne T, Phan TG, Chong W, Chandra RV, Bladin CF, Badve M, Rice H, de Villiers L, Ma H, Desmond PM, Donnan GA, Davis SM (2015). Endovascular therapy for ischemic stroke with perfusion-imaging selection. N Engl J Med.

[7] Jovin TG, Chamorro A, Cobo E, de Miquel MA, Molina CA, Rovira A, San Roman L, Serena J, Abilleira S, Ribo M, Millan M, Urra X, Cardona P, Lopez-Cancio E, Tomasello A, Castano C, Blasco J, Aja L, Dorado L, Quesada H, Rubiera M, Hernandez-Perez M, Goyal M, Demchuk AM, von Kummer R, Gallofre M, Davalos A (2015). Thrombectomy within 8 hours after symptom onset in ischemic stroke. N Engl J Med.

[8] Saver JL, Goyal M, Bonafe A, Diener HC, Levy EI, Pereira VM, Albers GW, Cognard C, Cohen DJ, Hacke W, Jansen O, Jovin TG, Mattle HP, Nogueira RG, Siddiqui AH, Yavagal DR, Baxter BW, Devlin TG, Lopes DK, Reddy VK, du Mesnil de Rochemont R, Singer OC, Jahan R (2015). Stent-retriever thrombectomy after intravenous t-PA vs. t-PA alone in stroke. N Engl J Med.

[9] Goyal M, Demchuk AM, Menon BK, Eesa M, Rempel JL, Thornton J, Roy D, Jovin TG, Willinsky RA, Sapkota BL, Dowlatshahi D, Frei DF, Kamal NR, Montanera WJ, Poppe AY, Ryckborst KJ, Silver FL, Shuaib A, Tampieri D, Williams D, Bang OY, Baxter BW, Burns PA, Choe H, Heo JH, Holmstedt CA, Jankowitz B, Kelly M, Linares G, Mandzia JL, Shankar J, Sohn SI, Swartz RH, Barber PA, Coutts SB, Smith EE, Morrish WF, Weill A, Subramaniam S, Mitha AP, Wong JH, Lowerison MW, Sajobi TT, Hill MD (2015). Randomized assessment of rapid endovascular treatment of ischemic stroke. N Engl J Med.

[10] Powers WJ, Derdeyn CP, Biller J, Coffey CS, Hoh BL, Jauch EC, Johnston KC, Johnston SC, Khalessi AA, Kidwell CS, Meschia JF, Ovbiagele B, Yavagal DR (2015). 2015 American Heart Association/American Stroke Association focused update of the 2013 guidelines for the early management of patients with acute ischemic stroke regarding endovascular treatment: a guideline for healthcare professionals from the American Heart Association/American Stroke Association. Stroke.

[11] Wahlgren N, Moreira T, Michel P, Steiner T, Jansen O, Cognard C, Mattle HP, van Zwam W, Holmin S, Tatlisumak T, Petersson J, Caso V, Hacke W, Mazighi M, Arnold M, Fischer U, Szikora I, Pierot L, Fiehler J, Gralla J, Fazekas F, Lees KR (2016). Mechanical thrombectomy in acute ischemic stroke: consensus statement by ESO-Karolinska stroke update 2014/2015, supported by ESO, ESMINT, ESNR and EAN. Int J Stroke.

[12] Powers WJ, Rabinstein AA, Ackerson T, Adeoye OM, Bambakidis NC, Becker K, Biller J, Brown M, Demaerschalk BM, Hoh B, Jauch EC, Kidwell CS, Leslie-Mazwi TM, Ovbiagele B, Scott PA, Sheth KN, Southerland AM, Summers DV, Tirschwell DL (2018). 2018 guidelines for the early management of patients with acute ischemic stroke: a guideline for healthcare professionals from the American Heart Association/American Stroke Association. Stroke.

[13] Goyal M, Menon BK, Coutts SB, Hill MD, Demchuk AM (2011). Effect of baseline CT scan appearance and time to recanalization on clinical outcomes in endovascular thrombectomy of acute ischemic strokes. Stroke.

[14] Han M, Choi JW, Rim NJ, Kim SY, Suh HI, Lee KS, Hong JM, Lee JS (2016). Cerebral infarct volume measurements to improve patient selection for endovascular treatment. Medicine.

[15] Li W, Li S, Dai M, Wang S, Xiong Y (2017). Comparisons of ASPECTS 5 and 6 for endovascular treatment in anterior circulation occlusive stroke. Interv Neuroradiol.

[16] Desilles JP, Consoli A, Redjem H, Coskun O, Ciccio G, Smajda S, Labreuche J, Preda C, Ruiz Guerrero C, Decroix JP, Rodesch G, Mazighi M, Blanc R, Piotin M, Lapergue B (2017). Successful reperfusion with mechanical thrombectomy is associated with reduced disability and mortality in patients with pretreatment diffusion-weighted imaging-Alberta Stroke Program Early Computed Tomography Score </=6. Stroke.

[17] Gilgen MD, Klimek D, Liesirova KT, Meisterernst J, Klinger-Gratz PP, Schroth G, Mordasini P, Hsieh K, Slotboom J, Heldner MR, Broeg-Morvay A, Mono ML, Fischer U, Mattle HP, Arnold M, Gralla J, El-Koussy M, Jung S (2015). Younger stroke patients with large pretreatment diffusion-weighted imaging lesions may benefit from endovascular treatment. Stroke.

[18] Inoue M, Olivot JM, Labreuche J, Mlynash M, Tai W, Albucher JF, Meseguer E, Amarenco P, Mazighi M (2014). Impact of diffusion-weighted imaging Alberta stroke program early computed tomography score on the success of endovascular reperfusion therapy. Stroke.

[19] Gautheron V, Xie Y, Tisserand M, Raoult H, Soize S, Naggara O, Bourcier R, Richard S, Guillemin F, Bracard S, Oppenheim C (2018). Outcome after reperfusion therapies in patients with large baseline diffusion-weighted imaging stroke lesions: a THRACE trial (mechanical thrombectomy after intravenous alteplase versus alteplase alone after stroke) subgroup analysis. Stroke.

[20] Barber PA, Demchuk AM, Zhang J, Buchan AM (2000). Validity and reliability of a quantitative computed tomography score in predicting outcome of hyperacute stroke before thrombolytic therapy. ASPECTS Study Group. Alberta Stroke Programme Early CT Score. Lancet.

[21] Nezu T, Koga M, Nakagawara J, Shiokawa Y, Yamagami H, Furui E, Kimura K, Hasegawa Y, Okada Y, Okuda S, Kario K, Naganuma M, Maeda K, Minematsu K, Toyoda K (2011). Early ischemic change on CT versus diffusion-weighted imaging for patients with stroke receiving intravenous recombinant tissue-type plasminogen activator therapy: stroke acute management with urgent risk-factor assessment and improvement (SAMURAI) rt-PA registry. Stroke.

[22] McTaggart RA, Jovin TG, Lansberg MG, Mlynash M, Jayaraman MV, Choudhri OA, Inoue M, Marks MP, Albers GW (2015). Alberta stroke program early computed tomographic scoring performance in a series of patients undergoing computed tomography and MRI: reader agreement, modality agreement, and outcome prediction. Stroke.

[23] Kim SK, Yoon W, Park MS, Heo TW, Baek BH, Lee YY (2016). Outcomes are not different between patients with intermediate and high DWI-ASPECTS after stent-retriever embolectomy for acute anterior circulation stroke. AJNR Am J Neuroradiol.

[24] Manceau PF, Soize S, Gawlitza M, Fabre G, Bakchine S, Durot C, Serre I, Metaxas GE, Pierot L (2018). Is there a benefit of mechanical thrombectomy in patients with large stroke (DWI-ASPECTS </= 5)?. Eur J Neurol.

[25] Lansberg MG, Straka M, Kemp S, Mlynash M, Wechsler LR, Jovin TG, Wilder MJ, Lutsep HL, Czartoski TJ, Bernstein RA, Chang CW, Warach S, Fazekas F, Inoue M, Tipirneni A, Hamilton SA, Zaharchuk G, Marks MP, Bammer R, Albers GW (2012). MRI profile and response to endovascular reperfusion after stroke (DEFUSE 2): a prospective cohort study. Lancet Neurol.

[26] Higashida RT, Furlan AJ, Roberts H, Tomsick T, Connors B, Barr J, Dillon W, Warach S, Broderick J, Tilley B, Sacks D (2003). Trial design and reporting standards for intra-arterial cerebral thrombolysis for acute ischemic stroke. Stroke.

[27] Grotta JC, Welch KM, Fagan SC, Lu M, Frankel MR, Brott T, Levine SR, Lyden PD (2001). Clinical deterioration following improvement in the NINDS rt-PA stroke trial. Stroke.

[28] Simonsen CZ, Schmitz ML, Madsen MH, Mikkelsen IK, Chandra RV, Leslie-Mazwi T, Andersen G (2016). Early neurological deterioration after thrombolysis: clinical and imaging predictors. Int J Stroke.

[29] Hacke W, Kaste M, Bluhmki E, Brozman M, Davalos A, Guidetti D, Larrue V, Lees KR, Medeghri Z, Machnig T, Schneider D, von Kummer R, Wahlgren N, Toni D (2008). Thrombolysis with alteplase 3 to 4.5 hours after acute ischemic stroke. N Engl J Med.

[30] Deguchi I, Dembo T, Yoshimura S, Sakai N, Okada Y, Kitagawa K, Kimura K, Hyogo T, Yamagami H, Egashira Y, Tanahashi N (2014). Relationship between magnetic resonance angiography-diffusion-weighted imaging mismatch and clinical outcome in endovascular treatment for acute ischemic stroke: subgroup analysis of the Recovery by Endovascular Salvage for Cerebral Ultra-acute Embolism--Japan Registry. J Stroke Cerebrovasc Dis.

[31] Daniere F, Lobotesis K, Machi P, Eker O, Mourand I, Riquelme C, Ayrignac X, Vendrell JF, Gascou G, Fendeleur J, Dargazanli C, Schaub R, Brunel H, Arquizan C, Bonafe A, Costalat V (2015). Patient selection for stroke endovascular therapy--DWI-ASPECTS thresholds should vary among age groups: insights from the RECOST study. AJNR Am J Neuroradiol.

[32] Berrouschot J, Sterker M, Bettin S, Koster J, Schneider D (1998). Mortality of space-occupying (‘malignant’) middle cerebral artery infarction under conservative intensive care. Intensive Care Med.

[33] Albers GW, Thijs VN, Wechsler L, Kemp S, Schlaug G, Skalabrin E, Bammer R, Kakuda W, Lansberg MG, Shuaib A, Coplin W, Hamilton S, Moseley M, Marks MP (2006). Magnetic resonance imaging profiles predict clinical response to early reperfusion: the diffusion and perfusion imaging evaluation for understanding stroke evolution (DEFUSE) study. Ann Neurol.

[34] Kawano H, Bivard A, Lin L, Ma H, Cheng X, Aviv R, O’Brien B, Butcher K, Lou M, Zhang J, Jannes J, Dong Q, Levi CR, Parsons MW (2017). Perfusion computed tomography in patients with stroke thrombolysis. Brain.

[35] Chen C, Parsons MW, Clapham M, Oldmeadow C, Levi CR, Lin L, Cheng X, Lou M, Kleinig TJ, Butcher KS, Dong Q, Bivard A (2017). Influence of penumbral reperfusion on clinical outcome depends on baseline ischemic core volume. Stroke.

[36] Lansberg MG, Cereda CW, Mlynash M, Mishra NK, Inoue M, Kemp S, Christensen S, Straka M, Zaharchuk G, Marks MP, Bammer R, Albers GW (2015). Response to endovascular reperfusion is not time-dependent in patients with salvageable tissue. Neurology.

[37] Nam HS, Lee KY, Han SW, Kim SH, Lee JY, Ahn SH, Kim DJ, Kim DI, Nam CM, Heo JH (2009). Prediction of long-term outcome by percent improvement after the first day of thrombolytic treatment in stroke patients. J Neurol Sci.

[38] Pu J, Wang H, Tu M, Zi W, Hao Y, Yang D, Liu W, Wan Y, Geng Y, Lin M, Jin P, Xiong Y, Xu G, Yin Q, Liu X (2018). Combination of 24-hour and 7-day relative neurological improvement strongly predicts 90-day functional outcome of endovascular stroke therapy. J Stroke Cerebrovasc Dis.

[39] Khatri P, Abruzzo T, Yeatts SD, Nichols C, Broderick JP, Tomsick TA (2009). Good clinical outcome after ischemic stroke with successful revascularization is time-dependent. Neurology.

[40] Khatri P, Yeatts SD, Mazighi M, Broderick JP, Liebeskind DS, Demchuk AM, Amarenco P, Carrozzella J, Spilker J, Foster LD, Goyal M, Hill MD, Palesch YY, Jauch EC, Haley EC, Vagal A, Tomsick TA (2014). Time to angiographic reperfusion and clinical outcome after acute ischaemic stroke: an analysis of data from the Interventional Management of Stroke (IMS III) phase 3 trial. Lancet Neurol.

[41] Nogueira RG, Jadhav AP, Haussen DC, Bonafe A, Budzik RF, Bhuva P, Yavagal DR, Ribo M, Cognard C, Hanel RA, Sila CA, Hassan AE, Millan M, Levy EI, Mitchell P, Chen M, English JD, Shah QA, Silver FL, Pereira VM, Mehta BP, Baxter BW, Abraham MG, Cardona P, Veznedaroglu E, Hellinger FR, Feng L, Kirmani JF, Lopes DK, Jankowitz BT, Frankel MR, Costalat V, Vora NA, Yoo AJ, Malik AM, Furlan AJ, Rubiera M, Aghaebrahim A, Olivot JM, Tekle WG, Shields R, Graves T, Lewis RJ, Smith WS, Liebeskind DS, Saver JL, Jovin TG (2018). Thrombectomy 6 to 24 hours after stroke with a mismatch between deficit and infarct. N Engl J Med.

[42] Albers GW, Marks MP, Kemp S, Christensen S, Tsai JP, Ortega-Gutierrez S, McTaggart RA, Torbey MT, Kim-Tenser M, Leslie-Mazwi T, Sarraj A, Kasner SE, Ansari SA, Yeatts SD, Hamilton S, Mlynash M, Heit JJ, Zaharchuk G, Kim S, Carrozzella J, Palesch YY, Demchuk AM, Bammer R, Lavori PW, Broderick JP, Lansberg MG (2018). Thrombectomy for stroke at 6 to 16 hours with selection by perfusion imaging. N Engl J Med.

[43] Costalat V, Lobotesis K, Machi P, Mourand I, Maldonado I, Heroum C, Vendrell JF, Milhaud D, Riquelme C, Bonafe A, Arquizan C (2012). Prognostic factors related to clinical outcome following thrombectomy in ischemic stroke (RECOST study). 50 patients prospective study. Eur J Radiol.

