# Supplementary material for: Acute ischemic stroke patients with diffusion-weighted imaging-Alberta Stroke Program Early Computed Tomography Score ≤ 5 can benefit from endovascular treatment: a single-center experience and literature review
Source: Neuroradiology. 2019 Feb 6;61(4):451–9. doi: 10.1007/s00234-019-02177-1 (PMC6431332; doi:10.1007/s00234-019-02177-1)
Supplement: Supplementary file 1 — (PDF 171 kb) [file 234_2019_2177_MOESM1_ESM.pdf]

Supplementary Table 1 Patient demographic and imaging information

Number	Gender	Age (years)	IS	AF	CHD	Hypertension	DM	Smoking
1	Male	53	Yes	No	No	Yes	No	No
2	Male	53	No	Yes	No	No	No	No
3	Male	85	No	Yes	No	Yes	No	No
4	Male	53	No	No	Yes	Yes	No	No
5	Female	53	No	No	No	No	Yes	No
6	Male	59	No	No	No	No	No	Yes
7	Female	65	No	Yes	No	Yes	No	No
8	Male	72	No	No	No	Yes	No	No
9	Male	54	No	No	No	No	Yes	Yes
10	Male	74	No	Yes	Yes	Yes	No	No
11	Male	83	No	Yes	No	Yes	Yes	No
12	Male	59	No	No	No	No	No	Yes
13	Male	78	No	Yes	Yes	Yes	No	No
14	Male	39	No	No	No	No	No	No
15	Female	45	No	No	No	No	No	No
16	Female	71	No	No	No	Yes	No	No
17	Female	82	Yes	No	Yes	Yes	Yes	No

18	Male	72	No	No	No	Yes	No	Yes
19	Male	83	Yes	Yes	No	Yes	No	No

IS=history of ischemic stroke; AF=atrial fibrillation; CHD=coronary heart disease; DM=diabetes mellitus.

Supplementary Table 2 Neurologic assessment, treatment protocol, and outcome

Number	Occlusion site	Lesion side	Baseline DWI-ASPECTS	Baseline hypoperfusion volume(ml)	Baseline infarction volume(ml)	Ratio of ASL-DWI mismatch	Intra-venous rt-PA	Stent implantation	Onset to rt-PA time(min)	Onset to femoral artery puncture time (min)	Onset to reperfusion time (min)	Admission NIHSS	Post-intervention NIHSS at 24 h	Post-treatment TICI	mRS at 3 months	Symptomatic hemorrhage
1	MCA	L	5	320	61.3	5.22	Yes	No	100	152	214	5	0	3	0	No
2	MCA	R	4	—	100	—	No	No	—	483	507	18	2	3	0	No
3	ICAi	R	2	552	283	1.95	Yes	No	66	128	155	15	7	2b	2	No
4	MCA	R	5	366	76.9	4.76	Yes	No	129	376	402	12	8	3	2	No
5	ICAe-MCA	R	3	413	233	1.77	No	Yes	—	125	256	15	11	2b	1	No
6	MCA	R	2	—	275	—	Yes	No	105	325	379	15	14	3	2	No
7	ICAi	R	5	218	58.7	3.71	Yes	No	120	485	515	19	15	3	2	No
8	ICAe	L	3	582	126	4.62	Yes	Yes	70	133	175	19	16	3	3	No
9	ICAi	L	5	221	10.1	21.9	No	No	—	335	410	17	17	3	3	No
10	ICAi	R	4	751	311	2.41	Yes	No	110	203	245	15	30	3	4	Yes
11	MCA	R	4	—	166	—	No	No	—	265	293	15	30	2b	6	No
12	ICAi	R	4	599	242	2.48	No	No	—	670	732	16	36	3	6	Yes
13	MCA	L	1	267	208	1.28	Yes	No	90	146	188	16	14	2b	2	No
14	MCA	L	5	260	88.3	2.94	No	No	—	319	350	18	6	3	0	No
15	ICAe	L	5	131	25.3	5.18	No	Yes	—	710	756	21	12	3	2	No
16	MCA	R	4	—	184.95	—	No	No	—	234	320	18	17	3	3	No

17	ICAi-MCA	R	5	260	188	1.38	No	No	—	232	325	16	9	2b	6	No
18	MCA	R	5	223	36.2	6.16	No	No	—	378	508	13	10	3	4	No
19	MCA	L	5	144	75.9	1.90	No	No	—	377	483	20	15	3	3	No

ICAi= intracranial internal carotid artery; ICAe=extracranial internal carotid artery; MCA= middle cerebral artery; R=right; L=left; NIHSS=the National Institutes of Health Stroke Scale; TICI= thrombolysis in cerebral infarction; mRS= modified Rankin Scale.
